# Charcot foot and ankle with osteomyelitis

**DOI:** 10.3402/dfa.v4i0.21361

**Published:** 2013-10-01

**Authors:** Ryan Donegan, Bauer Sumpio, Peter A. Blume

**Affiliations:** 1 Yale New Haven Hospital, New Haven, CT, USA; 2 Department of Surgery and Radiology, Yale School of Medicine, New Haven, CT, USA; 3 Department of Surgery, Anesthesia and Orthopedics and Rehabilitation, Yale School of Medicine, New Haven, CT, USA

**Keywords:** Charcot foot, osteomyelitis, diabetes mellitus, infection, neuropathy

## Abstract

This paper presents a review of the current literature discussing topics of Charcot osteoarthropathy, osteomyelitis, diagnosing osteomyelitis, antibiotic management of osteomyelitis, and treatment strategies for management of Charcot osteoarthropathy with concurrent osteomyelitis.

Charcot osteoarthropathy was first described over 140 years ago (1). Despite the time that has passed since the first publication in 1883, Charcot's joint disease of the foot and ankle remains a poorly understood and frequently overlooked complication of diabetes. Recognition, especially in the earliest stage, remains problematic with many cases going misdiagnosed even today. J.M. Charcot was the first to describe arthropathies, associated with tabes dorsalis (2). His early investigations into the tabetic arthropathies (1868) and his presentation, Demonstration of Arthropathic Affections of Locomotor Ataxy, at the Seventh International Medical Congress (1881), established the disease as a distinct pathological entity. Charcot and Féré published the first observations of the tabetic foot in the Archives de Neurologie in 1883. It was not until 1936, however, that W.R. Jordan established the association between neurogenic arthropathy of the foot/ankle and diabetes mellitus. Much of what is known about Charcot today initially came from studying patients with syphilis and leprosy, although diabetes has become the leading cause of the disorder.

Charcot osteoarthropathy is a relatively painless, progressive, and degenerative arthropathy of single or multiple joints caused by underlying neurological deficits, with peripheral joints most commonly affected. Charcot osteoarthropathy presents as a warm, swollen, erythematous foot and ankle, many times clinically indistinguishable from infection. Current estimates of Charcot osteoarthropathy prevalence ranges from 0.08% in the general diabetic population to 13% in high-risk diabetic patients (3). When severe infection is concurrent, morbidity and mortality rates can be as high as 35%, even when the infection is appropriately managed (3). Charcot osteoarthropathy usually occurs after 8–12 years from the diagnosis of diabetes, during the fifth and sixth decades, with men more frequently affected than women, and 30% incidence of bilateral involvement (3).

Diabetes may predispose to the occurrence of Charcot osteoarthropathy through a number of other mechanisms as well. Apart from the presence of neuropathy and possible osteopenia, these include the effects of advanced glycosylation end products, reactive oxygen species, and oxidized lipids, which may all enhance the expression of RANKL in diabetes (4). Other cases may be triggered by different causes of local inflammation, including previous ulceration, infection, or recent foot surgery. In this respect, the occurrence of an acute Charcot foot as a complication of osteomyelitis is increasingly recognized in people with diabetes.

In contrast to Charcot osteoarthropathy, osteomyelitis itself is an infection in bone. Infections can reach bone by traveling through the bloodstream or spreading from nearby tissue. Osteomyelitis can also begin in the bone itself if an injury exposes the bone to bacteria. People who have diabetes most often develop osteomyelitis in their feet as a result of foot ulcers (5). Twenty percent of diabetic patients develop osteomyelitis, which greatly increases the risk of lower extremity amputation (6).

## Differential diagnosis between Charcot osteoarthropathy and osteomyelitis

Charcot osteoarthropathy and osteomyelitis are difficult to diagnose when they occur concurrently, but it is important that diagnostic differentiation be made when addressing the pathological limb. Differentiation is important because treatment varies greatly depending on the pathology present. Charcot osteoarthropathy alone is treated differently from osteomyelitis or Charcot osteoarthropathy with osteomyelitis. If infection is present, regardless of the presence of Charcot osteoarthropathy, the osteomyelitis needs to be addressed and eradicated for successful treatment, which is accomplished in a staged strategy. Aside from osteomyelitis, differential diagnoses of Charcot osteoarthropathy include gout, deep vein thrombosis, and cellulitis. Chantelau (7) reported that 19 of 24 patients (80%) were misdiagnosed as having a sprain, osteomyelitis, Sudeck's atrophy, deep vein thrombosis, cellulitis, or rheumatoid arthritis instead of Charcot osteoarthropathy.

## Diagnostic difficulty of Charcot osteoarthropathy versus osteomyelitis

The difficulty in diagnostic differentiation of Charcot osteoarthropathy from osteomyelitis results from the two pathologies appearing very similarly with both clinical and imaging modalities of diagnosis. The presence of recent surgery, long-term antibiotic use, and concurrent Charcot osteoarthropathy all further complicate the diagnosis. As with any pathology, the same stepwise process is used, including clinical exam, laboratory test including complete blood count with differential (CBC with diff), erythrocyte sedimentation rate (ESR), C reactive protein (CRP), bone biopsy for histopathology and microbiology, imaging including x-ray, nuclear studies such as three-phase bone scan, WBC tagged studies (e.g. hexamethylpropyleneamine oxime, or HMPAO) in which focal uptake is consistent with osteomyelitis and diffuse uptake with cellulitis, and magnetic resonance imaging (MRI) (8). An emerging controversy is the accuracy of the ‘gold standard’, bone biopsy, in evaluating for osteomyelitis (9).

The usual presentation of Charcot osteoarthropathy involves a warm, swollen, erythematous foot or ankle in an insensate patient presenting almost identically to an acute soft-tissue infection (10, 11). A previous history of infections or ulcers can increase suspicion of a recurring acute or chronic infection. In the presence of an open wound, infection can be an easy diagnosis, but this does not exclude a concomitant Charcot process. Of note, there have been observed cases of Charcot osteoarthropathy that may have been triggered by cellulitis, osteomyelitis, and even synovitis (10, 12). Positive systemic signs of infection include leukocytosis, elevated CRP and ESR levels, and recent unexplained hyperglycemia; although unremarkable clinical tests do not exclude infection.

Although radiographs may not reveal bone or joint abnormalities during the inflammatory stage of Charcot osteoarthropathy or acute osteoarthropathy, these imaging studies in correlation with a clinical exam are one of the tools widely used to differentiate the pathologies (13). MRI exams are increasingly being used and recommended for diagnosing Charcot osteoarthropathy, especially at the earliest stage (13, 14). Although bone scintigraphy and white blood cell scans have been traditionally advocated for the differentiation and diagnosis of Charcot osteoarthropathy, there is clear evidence that MRI offers the highest diagnostic accuracy (15). Differentiating between acute Charcot osteoarthropathy and osteomyelitis is noted to be difficult due to similar signal intensity changes (16). Fortunately there are several MRI features, along with clinical correlation, anatomical distribution, and abnormal appearances that help distinguish these two diagnoses (15). In osteomyelitis, the pattern of bone marrow edema tends to involve a single bone with diffuse marrow involvement, where the pattern tends to be periarticular and subchondral in acute Charcot osteoarthropathy (15). Distribution of osteomyelitis usually has a focal involvement, the weight-bearing surfaces of the toes, metatarsal heads, or calcaneus (17), while acute Charcot osteoarthropathy usually has several joints/bones involved. Deformity is usually present with acute Charcot osteoarthropathy along with bone debris, where as osteomyelitis does not typically involve deformity unless there is an underlying Charcot osteoarthropathy process. The presence of soft-tissue infection, ulcerations, or sinus tract in the foot can improve the overall diagnostic accuracy for osteomyelitis (18), while MRI changes without these clinical findings may sway the diagnosis away from osteomyelitis.

## Osteomyelitis in patients with diabetes mellitus

A meta-analysis was conducted to determine the accuracy of historical features, physical examination, laboratory tests, and basic radiographic testing in diagnosing osteomyelitis in the lower extremity of diabetics (19). The conclusion from the study was that bone biopsy was the gold standard for diagnosis. In the meta-analysis, no studies were identified that addressed the utility of the history in the diagnosis of osteomyelitis. Other conclusions from the study included ulcer area larger than 2 cm^2^ and positive ‘probe-to-bone’ test were best clinical findings, ESR of more than 70 mm/h increases the probability of diagnosis, and abnormal plain radiographs double the odds of diagnosis. The study concluded that no single historical feature or physical examination reliably excludes osteomyelitis (19).

Surgical percutaneous bone biopsy specimen after a 14-day antibiotic-free period represents the gold standard of care for diabetic foot, with culture of bone specimens positive in 96% of patients (20). The sensitivity and specificity have been reported at 95% and 99%, respectively (21). On histopathological analysis, one can see signs of osteonecrosis, and on micropathological analysis, one can identify both acute and chronic processes. Acute infection will show infiltration of neutrophils, while chronic infection will show plasma cells and lymphocytes. When bone cultures are being obtained, it is recommended that antibiotics be withheld for at least 48 hours prior to culturing (22).

A total of 129 bacterial isolates were obtained from bone cultures with a mean of 1.6 isolates per patient (*Staphylococcus aureus*: 33%, Streptococci: 9%, Enterococci: 12%, Corynebacteria: 4%, Gram-negative bacilli: 20%, Anaerobes: 4%) (20). In percutaneous bone biopsy specimens, *S. aureus* was the common organism grown in culture (23). Wu et al. (24) performed a chart review to determine whether numerous factors affected the culture result of positive or negative cultures in histologically positive cases of osteomyelitis. The factors included a histological type of osteomyelitis, antibiotic therapy before biopsy, fever (temperature ≥38.0°C), elevated WBC count (≥10×10^3^ L), elevated ESR (≥10 mm/h), elevated CRP (≥6 mg/L), the size of the biopsy needle, and the amount of purulent fluid obtained at biopsy. Of the 41 cases of osteomyelitis, 34% had positive cultures. These factors did not have any significant association with positive or negative culture results. Wu concluded that the rate of positive culture results in histologically proven cases of osteomyelitis obtained from bone biopsies is low. Several studies suggest that 40–60% of histologically proven cases of osteomyelitis at surgery or biopsy are negative at culture (24).

The identification of a causative organism by culture both confirms osteomyelitis and allows tailoring of antimicrobial therapy, unfortunately cultures from samples obtained during surgery or biopsy are often negative. Current studies evaluating laboratory diagnosis of osteomyelitis are summarized in Table 1.

**Table 1:** Laboratory diagnostic studies

Weiner et al. (25)	Compared histology and microbiology diagnosis pedal osteomyelitis in diabetic patients	Results positive microbiologic and negative histological just as likely as negative microbiologic and positive histological	Microbiologic testing performed as well as histological testing in identifying pedal osteomyelitis diabetic foot
Senneville et al. (26)	Diagnostic value swab cultures compared to cultures of percutaneous bone biopsy for diabetic foot osteomyelitis	Bone and swab cultures identical for 17.4% patients, bone bacteria isolated from corresponding swab culture 30.4%. The overall concordance for all isolates 22.5%	Superficial swab cultures do not reliably identify bone bacteria
Senneville et al. (27)	Outcome diabetic patients suspicion osteomyelitis foot undergone percutaneous bone biopsy that yielded negative microbiological results		Diabetic patient with suspicion osteomyelitis and negative percutaneous bone biopsy, 1:4 develop osteomyelitis within 2 years of biopsy
Senneville at al. (28)	Compared needle puncture with concomitant transcutaneous bone biopsy	67.7% bone biopsy, 58% needle puncture, 96.7% swab positive culture results. *Staphylococcus aureus* most common type of bacteria that grew from bone samples, bone biopsy and needle puncture specimens identical 32.3%	Needle punctures compared with transcutaneous bone biopsies, do not identify bone bacteria reliably
Aragón-Sánchez et al. (29)	Investigated accuracy sequential combination probe-to-bone and plain x-rays diagnosing osteomyelitis	72.4% histologically proven osteomyelitis, 85.2% of which positive bone culture, sequential diagnostic sensitivity of 0.97, specificity of 0.92.	Clinicians can confidently diagnose diabetic foot osteomyelitis when both the probe-to-bone test and plain x-ray positive.
Lavery et al. (24)	Investigated probe to bone for identification of osteomyelitis	1,666 diabetic patients, probe to bone test positive predictive value 57% and negative predictive value 98%	Positive probe to bone test increases probability osteomyelitis slightly greater than 50%, negative probe to bone test strong predictor absence bone infection

There are many imaging modalities to aid in the non-invasive diagnosis of osteomyelitis, including plain radiographs, computer tomography (CT), MRI, sulfur colloid, WBC scan, positron emission tomography (PET) scan, single-photon emission computed tomography (SPECT/CT). MRI has gained considerable favor because it allows simultaneous evaluation of soft tissue and osseous structures for infective processes, as well as defining anatomical location of infected tissue with good accuracy and localization (30).

There have been many studies evaluating specificity and sensitivity of nuclear imaging. (99m)Tc-HMPAO-labeled WBC scintigraphy is a frequently used option for acute infection. In one small study, (99m)Tc-HMPAO labeled WBC scintigraphy was found to be true positive in six cases, true negative in six cases, and false negative in one patient who had a fever of unknown origin (31). Ubiquicidin 29-41 (UBI 29-41) is a synthetic antimicrobial peptide fragment reported to be highly infection-specific. UBI 29-41 was found to be consistent with osteomyelitis when the (99m)Tc-UBI 29-41 uptake was concordant with the (99m)Tc-MDP uptake (32). It was considered negative for osteomyelitis if there was no uptake of (99m)Tc-UBI 29-41 or if (99m)Tc-UBI 29-41 accumulated in an area not concordant with the abnormal uptake of (99m)Tc-MDP on the bone scan. In the latter case, a diagnosis of soft-tissue infection was made. The sensitivity, specificity and accuracy of (99m)Tc-UBI 29-41 scan in combination with three-phase bone scan for the diagnosis of osteomyelitis in diabetic foot was 100%. Accuracy for soft-tissue infection was also 100%. Maximum accumulation of the (99m)Tc-UBI 29-41 with maximum target to background activity was observed in the infectious foci at 30 min after injection (32).

Non-nuclear imaging such as MRI and PET scans also have a high specificity and sensitivity (30). Fluorodeoxyglucose positron emission tomography (FDG-PET) has been extensively investigated in differentiating acute infection from sterile post-surgical inflammatory processes. The results were mixed; while highly sensitive, its specificity with respect to distinguishing between acute infection and sterile inflammatory processes, including normal recuperative post-surgical healing, is limited (33). The conclusion was drawn, that in the complicated clinical context of acute post-surgical or post-traumatic infection, the diagnostic utility accuracy of FDG-PET is severely limited based on its focus on the increased glucose utilization that is generally characteristic of inflammatory processes.

From the studies, the best imaging for diagnosis of osteomyelitis in diabetic foot was (99m)Tc-UBI 29-41 scan in combination with three-phase bone scan, with sensitivity, specificity and accuracy 100%, also providing 100% for soft-tissue infection diagnosis. MRI is also a reliable option, with sensitivity of 100% (34). The limitations to an MRI exam include the presence of internal fixation devices, associated cost, and lack of available MRI equipment. Bone scintigraphy is highly sensitive, but lacks specificity in the diagnosis of Charcot osteoarthropathy (35). It is mainly used to rule out osteomyelitis in diabetic patients with open wounds that may or may not have bone destruction on radiographs. The determination to use bone scans should be based on ‘clinical suspicion’. However, when bone destruction is evident on radiographs without an open wound, then there is less need to undergo a three-phase 99technetium scan. Some authors recommend using leukocyte-labeled bone scans (111indium or 99mtechnetium HMPAO) to help exclude osteomyelitis (36). The diagnostic imaging modalities are summarized in Table 2.

**Table 2:** Imaging diagnostic studies

Aslangul et al. (37)	Investigated SPECT/CT coupled with bedside percutaneous bone biopsy when positive scan obtained	Sensitivity and specificity combined method 88.0 and 93.6%, respectively, PPV and NPV 91.7 and 90.7%, respectively.	Coupling of ^67^Ga SPECT/CT imaging and bedside percutaneous bone puncture accurate for diagnosing diabetic foot osteomyelitis
Bolouri et al. (38)	Suspected osteomyelitis or exacerbation known osteomyelitis investigated with CT and SPECT/CT.	Sensitivity, specificity and accuracy CT 77, 86 and 79%, and for SPECT/CT 100, 86 and 98%.	SPECT/CT significantly more accurate compared with CT.
Howe et al. (39)	Investigated if T1-weighted MRI features associated diabetic pedal osteomyelitis present in histologically proven cases non-pedal osteomyelitis.	93% cases demonstrated T1-weighted imaging features typical of pedal osteomyelitis with confluent region decreased signal intensity, hypointense, or isointense relative to skeletal muscle in a geographic pattern with medullary distribution.	Cases that did not demonstrate typical T1-weighted features predominantly secondary to hematologic mechanism of infection.
Kagna et al. (40)	Investigated FDG PET/CT for diagnosis osteomyelitis diabetic foot	FDG PET/CT sensitivity, specificity and accuracy of 100, 92 and 95% in a patient-based analysis and 100, 93 and 96% in lesion-based analysis	Foci sites of acute infection precisely localized with PET/CT allowing correct differentiation between osteomyelitis and soft-tissue infection
Morbach et al. (34)	Investigated bone scintigraphy to MRI for detecting osseous lesions	Inflammatory lesions detected 74.1% symptomatic regions by bone scintigraphy and 98.1% by MRI. Sensitivity of MRI compared to bone scintigraphy was superior in detecting lesions in the long bones of the thigh and the lower legs (100% vs. 78.4%, respectively).	MRI rather than plantar bone scintigraphy for detection chronic osteomyelitis

The many comorbidities associated with DM makes treatment of osteomyelitis often difficult. In diabetic patients, peripheral vascular disease can make antibiotic delivery inadequate and contribute to poor and slow healing of wounds, reduced renal function can also affect antibiotic choice. An impaired immune response frequently seen in patients with diabetes, can make early detection of infection difficult and then systemic fighting of infection inadequate, which can affect the onset, clinical course, and outcomes of osteomyelitis (41). In chronic osteomyelitis, sequestrum and involucrum form, which are infected devascularized bone fragments. Antibiotics do not penetrate devascularized bone; therefore, adequate surgical debridement, in addition to antimicrobial therapy, is necessary to cure chronic osteomyelitis.

## Antibiotic treatment of osteomyelitis

When treating osteomyelitis there are many considerations that affect treatment choices, such as antibiotic bone penetration, method of administration, and duration of therapy. Length of treatment for osteomyelitis depends upon clean margins, culture positive, and culture negative specimens. Antibiotic choice needs to be tailored to the organism isolated from infected bone.

The standard recommendation for treating chronic osteomyelitis is 6 weeks of parenteral antibiotic therapy (42). However, oral antibiotics have now become available that achieve adequate levels in bone. Oral and parenteral therapies achieve similar cure rates; however, oral therapy avoids risks associated with intravenous catheters and is generally less expensive, making it a reasonable choice for osteomyelitis caused by susceptible organisms. Antibiotic treatment for osteomyelitis is associated with moderate or severe adverse events in 4.8% of patients allocated oral antibiotics and 15.5% patients allocated parenteral antibiotics (43). The optimal duration of therapy for chronic osteomyelitis remains uncertain. There is no evidence that antibiotic therapy for more than 4–6 weeks improves outcomes compared with shorter regimens, and there is no evidence that prolonged parenteral antibiotics will penetrate the necrotic bone (42).

Antibiotic treatment of acute and chronic osteomyelitis should be considered as two distinct entities with regard to the choice of the most appropriate antibiotics and the need for surgery. Among the most recently available antibiotics, ertapenem and daptomycin are promising agents for the treatment of osteomyelitis due to resistant bacteria (44). Addition of adjunctive rifampin to other antibiotics may improve cure rates. Jeffcoate et al. (45) highlighted that recent studies have shown that antibiotics alone may apparently eliminate bone infection in many cases. There is also evidence that early amputation of infected digits is frequently non-curative. The ultimate test-of-cure is the lack of clinical relapse after the discontinuation of antimicrobials.

Chronic osteomyelitis is generally treated with antibiotics and surgical debridement but can persist intermittently for years with frequent therapeutic failure. Despite advances in both antibiotics and surgical treatment, the long-term recurrence rate remains at approximately 20–30% (43). Conterno and da Silva Filho (43) showed there was no statistically significant difference between the two groups, oral versus parenteral antibiotics, in the remission rate 12 or more months after treatment. Single trials with very few participants have found no statistically significant differences for remission or adverse events for the following three comparisons: parenteral plus oral (PO) versus parenteral only administration; two oral antibiotic regimens; and two parenteral antibiotic regimens (43). Limited evidence suggests that the method of antibiotic administration (oral versus parenteral) does not affect the rate of disease remission if the bacteria are sensitive to the antibiotic used. There is no solid evidence in the medical literature to support the continuous use of long duration antibiotic treatment for chronic osteomyelitis (46). A comparison of systemic antibiotic therapy studies is summarized in Table 3.

**Table 3:** Systemic antibiotic therapy

Rod-Fleury et al. (47)	Investigated duration of intravenous (IV) therapy on remission rates osteomyelitis	One week IV therapy same remission as 2–3 weeks or≥ 3 weeks. Greater than 6 weeks total antibiotic treatment equaled ≤6 weeks	Chronic osteomyelitis adult post-debridement antibiotic therapy beyond 6 weeks, or IV treatment longer than 1 week, did not show enhanced remission incidences.
Daver et al. (48)	Investigated *Staphylococcus* *aureus* cure rates comparing IV therapy to IV and PO antibiotic therapy	Overall apparent cure rate 74%; 69% IV group and 78% switch IV to PO antibiotics. Apparent cure rates similar regardless duration IV therapy: 83%<2 weeks, 72% 2–4 weeks, 75% 4–6 weeks, 66% ≥ 6 weeks.	MRSA infections responded poorly compared to MSSA (65% apparently cured versus 83%). However, 79% MRSA patients who received rifampin combinations, other than vancomycin and rifampin simultaneously were apparently cured.

There is also evidence for the effectiveness of local antibiotics in treating osteomyelitis. The primary benefit achieved with local antibiotic delivery vehicles is the ability to obtain extremely high levels of local antibiotics without increasing systemic toxicity. Antibiotic-loaded bone cement represents the current standard as an antibiotic delivery vehicle in orthopedic surgery. Composite biomaterials that simultaneously provide function of antibiotic delivery, and also contribute to the process of bone regeneration represent the most ideal class of local antibiotic delivery vehicles (49). Local antibiotic delivery with antibiotic loaded acrylic bone cement has been used extensively in the management of chronic osteomyelitis and implant-related infections. Self-made antibiotic loaded bone cement beads, which are cheaper and antibiotic specific, have been shown to elute less effectively than commercial antibiotic loaded cement beads (50). The commercial formulation of antibiotic cement produces an inhibition zone that is a bit larger and more regular than the manually mixed preparation. In clinical practice, low-dose antibiotic bone cement is often used. Although all carriers showed a burst release, low-dose antibiotic spacers showed little additional release after the first week, compared to the longer duration of antibiotic elution from commercial high dose antibiotic cement (51). Moojen et al. (51) cautioned the use of low-dose antibiotic bone cement for spacers because unsuccessful eradication of infection could result. A comparison of local antibiotic therapy studies is summarized in Table 4.

**Table 4:** Local antibiotic therapy

Chang et al. (52)	Evaluated antibacterial effects polymethylmethacrylate (PMMA) bone cements loaded with daptomycin, vancomycin, and teicoplanin against methicillin-susceptible *Staphylococcus aureus* (MSSA), methicillin-resistant *Staphylococcus aureus* (MRSA), and vancomycin-intermediate *Staphylococcus aureus* (VISA)	Regardless antibiotic loading dose, teicoplanin-loaded cements better elution efficacy and longer inhibitory periods against MSSA, MRSA, and VISA than cements with same dose vancomycin or daptomycin	For treatment *Staphylococcus aureus* infection, teicoplanin superior antibacterial effects
Shinsako et al. (53)	Investigated effect bead size and polymerization on PMMA bone cement vancomycin release	Cements loaded with higher dosages antibiotics showed longer elution periods	Beads which were smaller and had shorter polymerization time released more vancomycin

Peters et al. (54) presented a review article looking at the available evidence for accepted treatments of diabetic foot osteomyelitis, and found no significant differences in outcome associated with any particular treatment strategy. There was no evidence that surgical debridement of the infected bone is routinely necessary. They concluded culture and sensitivity of isolates from bone biopsy may assist in selecting properly targeted antibiotic regimens, but empirical regimens should include agents active against staphylococci. They also found no data to support the superiority of any particular route of delivery of systemic antibiotics or optimal duration of antibiotic therapy. No available evidence supports the use of any adjunctive therapies, such as hyperbaric oxygen, granulocyte-colony stimulating factor or larvae.

## Treatment strategies for Charcot osteoarthropathy with or without osteomyelitis

### Conservative treatment Charcot osteoarthropathy

The initial treatment for Stage 0 or Stage 1 Charcot osteoarthropathy is typically offloading in a total contact cast (TCC) (13). One specialty center's experience found that 60% of patients with midfoot Charcot osteoarthropathy had minimal deformity and were treated successfully without surgery (55). This finding emphasized that if acute Charcot osteoarthropathy is treated judiciously, achievement of a stable midfoot without incurring surgery or skin breakdown is possible. Although alternatives devices for immobilization and offloading have been studied, many clinicians consider the TCC as the treatment of choice (10, 11, 56–58).

Some clinicians have recommended an 8- to 12-week non-weightbearing immobilization in a TCC, while others have allowed weightbearing as tolerated from the start of treatment (13). Sinacore (59) found that the different anatomical locations affected by Charcot arthropathy would also affect healing times in TCC. Sinacore found that Charcot arthropathy of the hindfoot (mean, 97±16 days), midfoot (mean, 96±11 days), and ankle (mean 83±22 days) took significantly longer to heal in TCC than Charcot arthropathy of the forefoot (mean, 55±17 days). The total time of non-weightbearing TCC and the immobilization period in a weightbearing TCC or Charcot restraint orthotic walker (CROW) device may last up to 4–6 months (56). In a study of 70 neuropathic patients with 389 TCC changes, Guyton found a complication rate of 6% per cast (60, 61). The study concluded that TCC was a safe and reliable technique for offloading and immobilizing the neuropathic foot because of the predictable low rates of minor and reversible complications.

Bisphosphonates are commonly used anti-resorptive drugs against osteoporosis, Paget's disease, and other diseases with increased bone turnover, and have been used as a adjuvant pharmacologic therapy for acute Charcot osteoarthropathy, although the clinical efficacy of the treatment is controversial. Bisphosphonate treatment studies are summarized in Table 5.

**Table 5:** Bisphosphonate treatment

Jude et al. (62)	Compared saline vs. infused intravenous pamidronate, in random double-blinded placebo controlled study with acute Charcot osteoarthropathy patients	Found significant reductions in bone turnover markers, temperature, and pain symptoms	Significant findings in time to ambulation and time to radiographic consolidation not reported
Pakarinen et al. (63)	Investigated clinical effectiveness zoledronic acid in patients with diabetes and acute Charcot osteoarthropathy	Zoledronic acid group, median time for total immobilization 27 weeks, in placebo group 20 weeks	Zoledronic acid no beneficial effect on clinical resolution acute Charcot osteoarthropathy in total immobilization time, and may prolong time to clinical resolution
Pakarinen et al. (64)	Investigated effect immobilization and zoledronic acid on bone mineral density (BMD) changes during the treatment of acute midfoot Charcot osteoarthropathy	Significant fall BMD in placebo group at Charcot osteoarthropathy affected femoral neck, and Charcot osteoarthropathy free hip, with significant rise BMD in zoledronic acid group all measured areas Charcot osteoarthropathy free hip	Immobilization and off-loading does not lead to marked disuse osteoporosis in patients with acute Charcot osteoarthropathy after 6 months treatment
Bem et al. (65)	Randomized controlled study comparing bone turnover and temperature between study group received salmon calcitonin nasal spray daily with calcium supplementation and control group received only calcium supplementation	Study group significant reduction bone turnover compared with control group during 3-month follow-up	Advantage calcitonin may be direct impact on RANK-L/osteoprotegerin system, with fewer complications compared to bisphosphonate

Richard et al. (66) conducted a systematic review of the literature concerning the efficacy and safety of bisphosphonates in acute Charcot neuropathic osteoarthropathy. Bisphosphonates appeared to induce significant reductions in skin temperature and bone turnover markers compared with placebo, without serious adverse events. No studies showed that bisphosphonates shorten immobilization times, and no data is available regarding their long-term effects, along with efficacy regarding the occurrence of deformities and ulcerations. Moreover, some studies have suggested that bisphosphonates may lengthen the resolution phase of the disease. Richard et al. concluded that the data is too weak to support the use of bisphosphonates as a routine treatment for acute Charcot osteoarthropathy.

Of note, Black et al. (66) studied the use of bisphosphonates for postmenopausal osteoporosis and reported that serious atrial fibrillation occurred more frequently in the bisphosphonate group than in the placebo-controlled group. The significance of being a diabetic patient with multiple comorbidities are at greater risk of adverse events, and the use of bisphosphonates in acute Charcot osteoarthropathy patients must be carefully evaluated and all consequences must be examined fully.

### Treatment of Charcot osteoarthropathy with osteomyelitis

When formulating a treatment algorithm for Charcot osteoarthropathy, it is imperative to address all factors that will have an effect on outcome. First treating Charcot osteoarthropathy with concurrent osteomyelitis, the goal is as close to full eradication of osteomyelitis before final reconstruction takes place. The process of reconstruction of Charcot osteoarthropathy in the presence of an open wound, requires resection of the wound with elimination of osteomyelitis with antibiotic therapy (intravenous, oral, implantation of bone cement/antibiotic loaded beads/bone void filler with antibiotics), and exchange of bone cement or replacement of deficit with bone graft or primary arthrodesis with external fixation. Typically noted in patients with an open wound, negative pressure wound therapy with skin grafting may be necessary. For maintaining deformity correction achieved in reconstruction, Steinmann pins can be used for stabilization with compression from an Ilizarov external fixation for midfoot, hindfoot and/or ankle joints. Other options for surgical reconstruction include internal fixation ‘beaming’, external fixation, internal fixation, arthrodesis versus exostectomy, with the goal being complete fusion, although pseudoarthrosis can create a stable lower extremity allowing for ambulation and reduced risk of ulceration/re-ulceration.

Aragón-Sánchez et al. (67) presented treatment of diabetic foot osteomyelitis, where conservative surgical procedures were performed, defined as no amputation of any part of the foot. They found conservative surgery without local or high-level amputation is successful in almost half of the cases of diabetic foot. The risks of failure in the case of conservative surgery were exposed bone, the presence of ischemia and necrotizing soft tissue infection. Less than 50% cure rate is not acceptable for the treatment of diabetic pedal infections, highlighting the need for surgical intervention. A severe diabetic foot infection carries a 25% risk of major amputation (68). The overall strategy for surgically managing a severe diabetic foot infection is infection control through aggressive and extensive surgical debridement, a comprehensive vascular assessment with possible vascular surgery and/or endovascular intervention, and soft tissue and skeletal reconstruction after infection is eradicated to obtain wound closure and limb salvage.

Pinzur et al. (69) conducted the largest study to date, involving 73 cases of lower extremity Charcot osteoarthropathy with osteomyelitis, in which he used a single stage procedure for treatment. The first step of the surgery involved radical resection of clinically infected bone. Tissue cultures from the resected bone were used to guide parenteral antibiotic therapy. Sufficient bone was removed to allow correction of the deformity to a plantigrade position. Large smooth percutaneous pins were used for provisional fixation. Maintenance of the surgically obtained correction was achieved with a three-level preconstructed static circular external fixator. Wounds were loosely reapproximated when possible and managed with adjuvant dressings and wound care when they could not be closed. Patients were all treated with culture-specific parenteral antibiotic therapy that was administered and monitored by an infectious disease co-management consultation service. The infectious disease consultant made both the choice and duration of antibiotic therapy. The circular external fixator was maintained for a period of 8 weeks in patients with deformity in the foot and a minimum 12 weeks when the ankle was involved. Following removal of the external fixator, patients were managed in a weight-bearing TCC for 4–6 weeks, followed by a commercially available diabetic fracture boot. Patients were transitioned to therapeutic footwear consisting of commercially available depth-inlay shoes and custom accommodative foot orthoses. Using this protocol, Pinzur was able to achieve 95.7% limb salvage with ambulation in commercially available therapeutic footwear. There have been numerous studies utilizing parts or all of this protocol, with high success rates for treating Charcot osteoarthropathy with osteomyelitis of both the midfoot and ankle.

Farber et al. (70) reported on 11 patients with midfoot Charcot osteoarthropathy and ulceration, which underwent operative debridement, corrective osteotomy, external skeletal fixation and culture-directed antibiotic therapy as a limb salvage procedure. Patients were transitioned from the external fixator to TCC, and all subsequently progressed to therapeutic footwear in 12–49 months of follow-up.

In one study, 26 diabetic adults had operative correction of nonplantigrade Charcot osteoarthropathy midfoot deformity at the midfoot level (71). Correction was maintained with a neutrally applied three-level ring external fixator. Fourteen had open wounds with underlying osteomyelitis. Surgery included Achilles tendon lengthening, excision of infected bone, correction of the multiplanar deformity, and culture-specific parenteral antibiotic therapy. Twenty-four of 26 patients were ulcer- and infection-free and able to ambulate with commercially available depth-inlay shoes and custom accommodative foot orthoses.

In another study, eight patients with diffuse ankle osteomyelitis were treated by resection of all infected tissue and hybrid-frame compression arthrodesis (72). Fusion of eight ankles and four subtalar joints was attempted. All patients received 6 weeks of intravenous antibiotics. Open wounds were treated with wound vacuum assisted closure devices until closure was achieved. Frames were removed at 3 months and walking casts were applied for 1–2 further months. Ankle sepsis was eradicated in all patients. Seven out of eight ankles fused at an average of 13.5 weeks. At an average 3.4-year follow-up, none of the seven fused ankles required further surgery.

Paola et al. (73) conducted a prospective study evaluating limb salvage through surgical treatment of osteomyelitis of the midfoot or the ankle and stabilization with external fixation. Out of 45 patients with Charcot osteoarthropathy and osteomyelitis who underwent debridement and attempted fusion with an external fixator, 39 patients healed using emergent surgery to drain an acute manifestation of the infection while maintaining the fixation for an average of 25.7 weeks.

Pawar et al. (74) presented a study looking at patients with Charcot osteoarthropathy whose infected ankles were treated with a retrograde, antibiotic-coated, locked intramedullary nail. In all patients, bony union was achieved and infection was eradicated, with an average time for radiological healing of 4.1 months, and no cases of hardware failure.

Pinzur et al. (11) presented 49 feet with midfoot neuropathic foot deformities that were followed for an average of 3.6 years. Twenty-six of the feet initially presented for care with open ulcers and/or chronic osteomyelitis. Treatment included debridement of the infected bone and surrounding soft tissues, exostectomy and partial excision of the deformed midfoot combined with boney stabilization and attempted arthrodesis. All surgical patients were managed postoperatively with long-term custom accommodative bracing. All but one of the patients remained ambulatory, and none required below-knee amputations.

Adjunctive surgical procedures are also used to increase surgical effectiveness for treating Charcot osteoarthropathy with osteomyelitis. In one study, 20 patients with Charcot osteoarthropathy of the foot and ankle were treated with an Ilizarov external fixator (75). Each patient had an open lengthening of the tendo achilles with excision of all necrotic and loose bone from the ankle, subtalar and midtarsal joints when needed. The resulting defect was packed with corticocancellous bone graft harvested from the iliac crest and an Ilizarov external fixator was applied. Arthrodesis was achieved after a mean of 18 weeks, with healing of the skin ulcers. Every patient was able to resume wearing regular shoes after a mean of 26.5 weeks.

Diabetic patients with Charcot osteoarthropathy are complex patients, with many co-morbidities other than osteomyelitis. The introduction of the hospitalist co-management model represents an opportunity to improve care. Pinzur et al. (76) conducted a study investigating the outcomes of diabetic patients undergoing treatment of osteomyelitis and Charcot osteoarthropathy reconstruction after being enrolled in an academic medical center hospitalist-orthopedic surgery co-management patient care program. While the overall observed-to-expected cost of care remained virtually unchanged, the positive impact of the study model revealed an increased positive effect on the more severely affected severity of illness and risk of mortality patients. The results of this study suggest that a proactive, cooperative, co-management model for the perioperative management of high-risk patients undergoing complex surgery can improve the quality and efficiency metrics associated with the delivery of service to patients.

Charcot osteoarthropathy healing is dependent upon many factors, including hemoglobin A1c, creatinine, overall co-morbidities, underlying systemic arthropathies such as rheumatoid and lupus, chronic prednisone use, end-stage renal disease on hemodialysis, which is why a team approach is crucial for successful outcomes.

Ultimately, there has not yet been agreement on protocol for treating Charcot osteoarthropathy of the lower extremity with or without osteomyelitis. The most current literature for treatment of Charcot osteoarthropathy and osteomyelitis highlights the lack of consensus, although does show some commonality between approaches.

## Conclusion

The process of treating Charcot osteoarthropathy with concurrent osteomyelitis is an extremely involved and long-term process, involving multiple surgeries, antibiotics, extended periods of non-weightbearing and immobilization, external fixation. It is a huge undertaking, and probably one of the most involved and taxing physical and mental undertakings a patient can do, all without any guarantee of correction or prevention of recurrence.

Questions that need to be addressed include length of treatment, what is the impact of long-term immobilization, is reconstruction of Charcot osteoarthropathy with osteomyelitis a better treatment than primary amputation? Perhaps the first study that needs to be performed is functional scores to compare below-knee amputation with reconstruction to determine if this undertaking is beneficial for the patient.

In summary, this paper has presented current literature discussing topics of Charcot osteoarthropathy, osteomyelitis, diagnosing osteomyelitis, antibiotic management of osteomyelitis, and treatment strategies for the management of Charcot osteoarthropathy with overlying osteomyelitis.
